# Artificial intelligence in assessing progression of age-related macular degeneration

**DOI:** 10.1038/s41433-024-03460-z

**Published:** 2024-11-18

**Authors:** Sophie Frank-Publig, Klaudia Birner, Sophie Riedl, Gregor S. Reiter, Ursula Schmidt-Erfurth

**Affiliations:** Laboratory for Ophthalmic Image Analysis, Department of Ophthalmology and Optometry, Medical University of Vienna, Vienna, Austria

**Keywords:** Retinal diseases, Predictive markers, Macular degeneration

## Abstract

The human population is steadily growing with increased life expectancy, impacting the prevalence of age-dependent diseases, including age-related macular degeneration (AMD). Health care systems are confronted with an increasing burden with rising patient numbers accompanied by ongoing developments of therapeutic approaches. Concurrent advances in imaging modalities provide eye care professionals with a large amount of data for each patient. Furthermore, with continuous progress in therapeutics, there is an unmet need for reliable structural and functional biomarkers in clinical trials and practice to optimize personalized patient care and evaluate individual responses to treatment. A fast and objective solution is Artificial intelligence (AI), which has revolutionized assessment of AMD in all disease stages. Reliable and validated AI-algorithms can aid to overcome the growing number of patients, visits and necessary treatments as well as maximize the benefits of multimodal imaging in clinical trials. Therefore, there are ongoing efforts to develop and validate automated algorithms to unlock more information from datasets allowing automated assessment of disease activity and disease progression. This review aims to present selected AI algorithms, their development, applications and challenges regarding assessment and prediction of AMD progression.

## Introduction

Age-related macular degeneration (AMD) causes irreversible vision loss in millions of people worldwide. Estimations expect 288 million people affected by AMD in 2040 [1]. With an increasing number of people in the elderly generations and novel intravitreal therapies for geographic atrophy (GA) [2, 3], the vast number of examinations and injections could become a burden for ophthalmologists limiting time for each patient as well as for health care providers. Simultaneously, multimodal imaging techniques and knowledge with respect to the visualized biomarkers are constantly improving. The amount of relevant information captured per patient is, therefore, expanding. Thus, automated analysis of data will become key in the future management of AMD. AI-based algorithms provide reliable and precise quantification of retinal features in a fraction of the time a human examiner would need, providing more time for doctor-to-patient interactions.

Patients affected from early stages of the disease, including early and intermediate AMD (iAMD), mostly experience no symptoms until progression to advanced stages, GA, and neovascular AMD (nAMD). Conversion rates to advanced stages [4] and progression speed vary highly between patients [5], hence accurately predicting progression is of utmost importance in preventing vision loss and optimizing monitoring intervals. Patients with nAMD benefit from therapy with anti-vascular endothelial growth factors (anti-VEGF) and, recently, agents combined with angiopoetin-2 inhibitors [6]. For GA, on the other hand, two novel intravitreal therapeutics have been approved in the US by the regulatory authorities in 2023. These new agents based on complement inhibition slow down the growth of atrophic lesions [2, 3]. Due to the lack of any noticeable, immediate functional benefit to the patient, paired with the need of regular intravitreal injections, compliance could become problematic. Prediction models can visualize an individual’s estimated disease progression, which can be the key to increase patient motivation in order to initiate or continue treatment. Multimodal imaging is often required in both atrophic and nAMD, specifically at the stage of diagnosis. Nevertheless, optical coherence tomography (OCT) is the key imaging modality for monitoring AMD due to its fast and non-invasive three-dimensional high-resolution visualization of the retina. Therefore, major developments of AI in retina focus on biomarker quantification from OCT volumes [7].

**Fig. 1:**
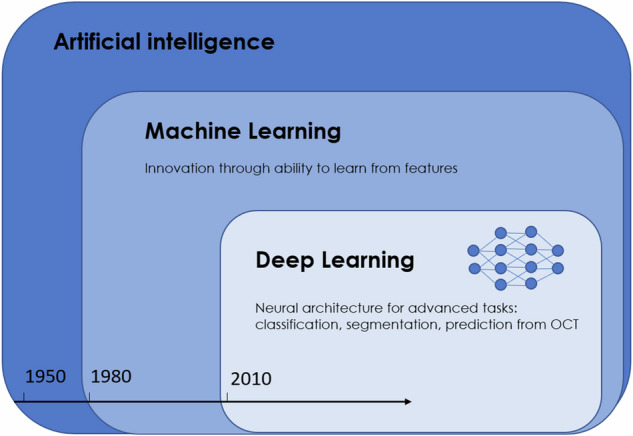
Ongoing developments in Artificial Intelligence. Adapted from *Deep learning in ophthalmology*: *The technical and clinical considerations*, Ting et al. [130].

AI has been introduced to multiple fields of medicine, however, ophthalmology is considered to have the widest scope of algorithms available since it heavily relies on imaging. AI models have been developed to analyze retinal imaging data for classification, segmentation, and prediction, supporting clinicians, and researchers, first and foremost for diabetic retinopathy, retinopathy of prematurity, and AMD. Multiple AI-based methods have been developed and applied (Fig. 1) [8].

The classic form of AI is machine learning (ML), where algorithms learn from biomarkers or *features* derived directly from the data. During development, there are at least two steps involved. First, in the training dataset, the model learns to connect certain features indicated by human experts to *labels* (e.g., classification or segmentation) or values (e.g., prediction models), referred to as supervised learning. For example, a classifier needs to learn to recognize drusen in order to diagnose early and intermediate AMD, which would be a practical screening tool. In the second step, the algorithm is tested in an unacquainted (part of the) dataset to assess the performance. Further steps can be initiated to enhance the performance or validate the algorithm. These models are dependent on appropriate and meaningful features for each label, which directly affects the model performance. The basis of ML is artificial neural networks, which are computing systems imitating the central nervous system by transforming signals in layers from input to output [8].

Such artificial neural networks have been further refined to deep neural networks (DNN) implementing multiple layers adding to the complexity of the model. In this technique, the signal is transformed into more abstract and higher-level forms while using fewer artificial neurons compared to shallow artificial neural networks. Furthermore, unlike ML, the performance of deep learning (DL) algorithms improves with the size of the dataset. With increasing computational power and large datasets, DNNs can be trained and applied in a short amount of time outperforming the original artificial neural networks. This initiated the introduction of DL, where neural networks are used not only as classifier, but also as feature extractor at the same time. Therefore, a single DNN can simultaneously learn to extract characteristic features and classify based on these biomarkers. The benefit of this method is a direct extraction of features without human-engineered feature-extraction, also called unsupervised or end-to-end learning. For imaging data, a specific type of DNN, convolutional neural networks (CNN), have proven particularly suitable. They resemble connectivity patterns of the mammalian visual cortex. First, a mathematical filter (convolution) is added as layer resulting in single neurons assessing only a subfield of the image. Then, multiple layers of this network are stacked together resulting in more sophisticated feature detectors. These detectors are adjusted to recognise characteristic patterns during training in order to fulfill the required task [8]. Besides classification, CNNs can be trained to segment and quantify retinal features in a pixelwise manner such as fluid, drusen, and retinal layers. CNN-based classifiers can learn from color fundus photography (CFP) or OCT to diagnose AMD [9], and differentiate between nAMD and GA [10, 11]. These tools could be used for screening in the inclusion phase of clinical trials or in regions of limited medical care for more efficient referrals. Furthermore, AI can be used to rule out differential diagnoses of GA using multimodal imaging [12, 13]. Besides classification, CNNs can be trained to segment and quantify retinal features in a pixelwise manner such as fluid, drusen, and retinal layers. Due to the importance of layer segmentation for the assessment of several tasks relevant for disease progression, such as the integrity of outer retinal layers, a convolutional network, termed U-Net has been adapted particularly for this task [14, 15]. Further tools in the field of AI have been introduced, such as transfer learning, which enables the use of small datasets for development by fine-tuning pre-existing CNNs trained on large datasets [16]. Other methods have been used to develop automated algorithms, such as recurrent neural networks (RNNs), and generative adversarial networks (GANs) [17]. RNNs are well-suited for sequence labeling and for prediction tasks, as they save past input within the network, which is then considered during output production [18]. GANs consist of a generator and a discriminator, two networks working together to synthesize high-quality data. A benefit of this technique is the independence from imbalanced datasets. With this method, for OCT-based assessment of AMD, no manual labelling is necessary due to augmented data for training. GAN-generated retinal images can reach a level of being indistinguishable from real ones [19]. Furthermore, GAN-based synthesized images aid in denoising achieving super-resolution in OCT images [20].

In conclusion, AI is capable of automatically assessing images from multiple imaging modalities and devices within a fraction of the time compared to human assessment. These models are capable of fulfilling multiple tasks, such as image recognition for classification tasks and more complex models can even predict future progression. Moreover, further benefits include excellent reproducibility and the performance of some algorithms can even exceed the performance of human graders. Due to the highlighted need for automated analysis and a multitude of revolutionary developments in the field of AI in AMD, we aim to present a comprehensive review of automated algorithms capable of assessing and predicting progression for AMD.

## Methods

An assessment of the literature was performed between December 2023 and March 2024 using Pubmed, Google Scholar, Scopus, and Medline without any restrictions. Searched keywords included AMD, intermediate AMD, GA, neovascular AMD, AI, machine learning, deep learning, optical coherence tomography, multimodal imaging, progression, and prediction.

## Results

### Early and intermediate AMD

Early stages of AMD are characterized by drusen and pigmentary alterations resulting in minor to no visual complaints. Disease progression to advanced stages with relevant vision loss varies highly between individuals. Historically, classification of AMD and diagnosis of iAMD was based on CFP [21]. Recent technological advances in OCT technology led to a shift towards OCT, as it provides detailed cross-sectional visualization of important features beneficial for early detection and monitoring of disease progression. This led to breakthroughs in volumetric quantifications of pathognomonic iAMD biomarkers [22], such as drusen, which accumulate in the sub-retinal pigment epithelium (RPE) layer [21].

Due to limited symptoms in early stages of the disease, the detection of drusen often occurs as incidental finding. However, multiple algorithms have been trained to classify AMD automatically on multiple imaging modalities including CFP [9], fundus autofluorescence (FAF) [10], near-infrared reflectance (NIR) imaging, OCT, and combinations thereof [11, 23], which can be useful screening tools for the inclusion of patients in longitudinal studies.

#### AI in assessment of biomarkers for progression of iAMD

The assessment of iAMD progression remains challenging due to subtle and subclinical symptoms and slow progression, reflected in the lack of clinical endpoints for iAMD trials. Until now, best-corrected visual acuity (BCVA), FAF-based progression to advanced AMD, microperimetry-based point-wise sensitivity changes [24], drusen area [25], or drusen volume [26] have been used as outcome measures. However, no validated clinical endpoints for iAMD trials exist. Several early atrophic features have been identified on OCT, including nascent GA or incomplete RPE and outer retinal atrophy. Both have been defined as precursors of GA. The OCT-based correlation to GA, as defined by the CAM group has been termed complete RORA. Future clinical trials for development of novel therapeutic targets in iAMD would benefit from more reliable trial endpoints by assessment and definition of subclinical changes on OCT. The PINNACLE study and the MACUSTAR consortium aim to establish such novel validated functional and structural endpoints in iAMD [27, 28]. Multiple indicators for conversion to GA have been identified in OCT: Wedge-shaped subretinal hyporeflectivity, RPE attenuation and disruption, drusenoid RPE detachment, RPE thickening [29], hyperreflective foci (HRF) [29], subretinal drusenoid deposits (SDD) [30, 31], outer plexiform layer (OPL) subsidence, outer retinal layer thickness reduction [32], PR atrophy [33], hyporeflective cores in drusen, and high drusen volume [34]. Pfau et al. demonstrated drusen regression with thinning of the outer PR layers prior to integrity loss, followed by RPE loss [33]. For clinical diagnosis and management, drusen remains the benchmark biomarker in early stages of AMD. Therefore, various groups developed and implemented ML and DL-based algorithms for automated layer segmentation and subsequent drusen volume quantification in recent literature to demonstrate their role in AMD progression [22, 35]. Moreover, analyses of drusen morphology demonstrated an association between drusen and overlying ellipsoid zone (EZ) and interdigitation zone (IZ) thinning and reduced retinal sensitivity in microperimetry (MP) [36, 37].

Integrity of outer retinal layers with focus on the metabolically active EZ layer and the hyporeflective outer nuclear layer (ONL) as part of the inner segment of the photoreceptor, has been defined as another quantifiable subclinical biomarker in iAMD. Due to the importance of outer retinal integrity, and the infeasibility of assessing these small changes by simple clinical evaluation, algorithms have been developed. Saßmannshausen et al. described an association of increased disease severity with automatically measured reduced relative reflectivity of the EZ in iAMD. Challenges during the development of this algorithm include a limited number of patients with early AMD as well as missing follow-up data. Furthermore, SDD were not excluded when segmenting the EZ layer which might lead to falsely increased reflectivity signals. The impact of this novel biomarker needs to be evaluated in further studies [38]. In pioneering work, Orlando et al. established automated EZ-thickness measurements by developing a validated U-Net-based CNN algorithm for segmentation of the EZ and the IZ in each B-Scan [39]. Automated quantification of EZ-thickness has been established as another prognostic biomarker by various groups for multiple devices, which is desirable for clinical implementation. Research groups frequently explore multiple neural architectures before determining the final model due to large differences in the performance for future applications in external datasets [40, 41]. Concurrently, Pfau et al. implemented automated quantification of the ONL-thickness as a prognostic biomarker [33], while there are recent efforts to quantify and detect OPL subsidence in an automated manner [42](Aresta et al. under review). The difficulty in ONL and OPL segmentations lies within the Henle fiber layer, which presents with the same reflectivity as the OPL. In patients with AMD, this effect might derive from dyslamination, which is characterized by disorder within these layers and migrating photoreceptor nuclei, however, further clinicopathologic studies are necessary to confirm these theories [43]. Additional to thinning of EZ and the ONL, quantification of HRF has been identified as a potential biomarker for disease progression. In AMD histology, HRF are described as activated ‘intraretinal’’ RPE, migrating anteriorly [44] and they have been associated with conversion from earlier stages of AMD to GA [45]. Schlegl et al. introduced a validated DL-algorithm based on a residual U-NET to automatically detect and quantify HRF in a reliable manner [46]. However, retinal vessels on OCT may be falsely marked as HRF, whereas a registration algorithm [46] combined with a component-filtering algorithm can be used to remove false-positive vessels minimizing this effect [47]. Besides HRF, SDD have been associated with a high risk for outer retinal atrophy with fast GA growth and developing type 3 macular neovascularisation (MNV) [31, 48]. Therefore, efforts in the development of automatic segmentations of SDD on OCT have been made [49]. However, low inter-reader agreement of SDD segmentations further highlights the difficulty of this task [50]. Still, pivotal CNN-based algorithms were developed for automated SDD segmentation in spectral domain OCTs (SD-OCT) [51, 52], while implementation to external datasets was not yet introduced.

Since no effective therapy is currently available, AI-based phenotyping could aid categorization of high-risk and low-risk groups for more personalized and therefore lower resource monitoring intervals. Predicting the onset of conversion to late AMD is currently relevant in clinical routine with respect to the early detection of treatable late stages of the disease.

#### Prediction of conversion to advanced AMD

**Fig. 2:**
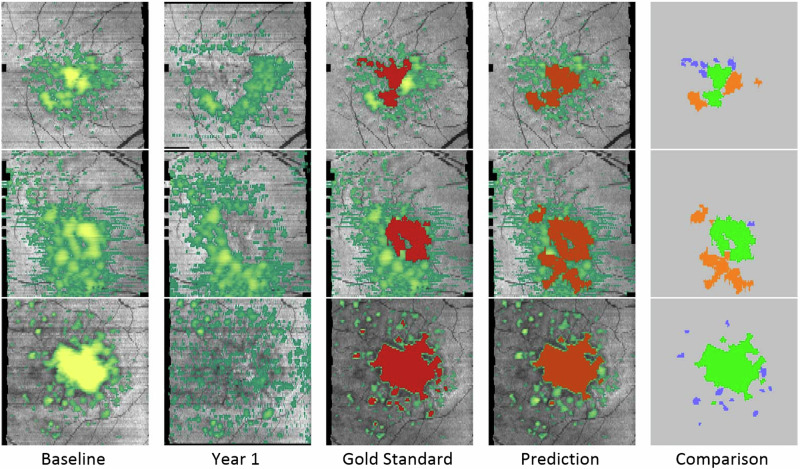
Machine-learning-based predictive model for drusen regression. En-face map of drusen volume at baseline and year 1 (first columns) and prediction of drusen regression by machine learning (fourth column) and comparison to “real” change (third and fifth column) with permission from Bogunovic et al. [22].

Next to environmental factors, demographics, and genetics influencing disease progression [53], imaging has provided multiple biomarkers preceding conversion to advanced AMD. During progression of AMD, drusen show specific dynamic processes. They change in size, fuse together, or regress. Drusen regression can occur without any damage to the retina, although eventually it might lead to atrophy or MNV [54] at the same location [53, 55]. Therefore, drusen dynamics is an important feature regarding assessment and prediction of AMD progression. Garzone et al. demonstrated that disease progression can be assessed automatically by observing drusen volume and related changes over time. Limitations during the development of this algorithm included a relatively small sample size, the lack of external validation and limited repeatability for Spectralis OCT, while this algorithm shows promising performance for Cirrus OCT [56]. Furthermore, Bogunovic et al. developed an ML-based predictive model based on layer segmentations of the outer retina and HRF (Fig. 2). The impact of drusen height, thickness, area, and volume as well as outer retinal layer thickness and attenuation was assessed in longitudinal data. In this study, SDD were excluded as prognostic biomarker due to unreliable automated segmentation. The predictive model achieved an AUC of 0.75 for prediction within the first two years. One limitation of this study is a relatively small sample size. Therefore, the reported findings should be generalized with caution [22]. Vogl et al. published a reference frame for spatial and longitudinal OCT analysis in the HARBOR trial and identified ONL and EZ thinning as morphologic markers associated with atrophy development and choroidal changes linked to MNV onset. Assessing the area posterior to the RPE, i.e., the choroid, in SD-OCT images is a particularly challenging task due to absorption of the light by RPE and an associated low signal-to-noise ratio. Therefore, texture descriptors were added for analysis of this area. However, the interpretation of these texture features remains limited due to a lack of physical correlates. Alternatively, swept-source OCT or OCT angiography could be used to examine choroidal patterns [57]. To characterize and validate biomarkers for disease progression and to develop predictive risk models, the PINNACLE consortium collected real-world data from over 400,000 images including OCT from patients with AMD. Based on this data containing various stages of AMD and healthy eyes, an algorithm predicting disease progression is being developed using supervised and unsupervised ML. Furthermore, a DL approach using data from the PINNACLE trial based on a two-stage CNN classifier learned to distinguish between normal eyes and all stages of AMD achieving high accuracy [58]. This tool could be used for screening in isolated regions of the world or to identify patients with early and intermediate AMD for inclusion in larger study cohorts. This would be beneficial for patient recruitment, as patients with early AMD or intermediate AMD are frequently incidental findings due to late functional impact of the disease. Concomitantly, various groups demonstrated the effect of multifactorial determinants affecting disease progression in iAMD [59–62]. Furthermore, recent efforts were made using innovative self-supervised learning methods, such as Morph-self-supervised learning, in which morphological changes between OCT scans of different timepoints is simulated. This could provide further insight into progression risk and has been studied for the progression from iAMD to nAMD (Chakravarty et al. under review). These insights can be beneficial for clinicians estimating an appropriate time-point for check-ups at the time of conversion in order to start treatment as early as possible.

An ML approach by Ajana et al. was trained on data from the Rotterdam Study. Here, a method called bootstrap least absolute shrinkage and selection operator was used, where the relevance of each feature is adapted automatically. In this study, a large variety of environmental factors were considered leading to a small number of events limiting the performance of the algorithm [63]. Furthermore, it would require a certain amount of time to determine all required environmental risk factors including the genetic risk score, the Mediterranean diet score or the patient’s education for optimal performance of this algorithm. Another ML approach predicting the conversion to nAMD or GA in fellow eyes of patients in the HARBOR trial learned from OCT-based features, demographics, and genetic profiles. Thickness maps of the ONL, the EZ, and RPE layers, drusen, SDD, and HRF were used as structural features for training of this algorithm, providing a hazard ratio for the risk of conversion as output. In this study, converters to GA show global ONL thinning, RPE, and EZ thinning, and HRF in the ONL, whereas nAMD-converters present thickening of the RPE-drusen complex, increased drusen area, HRF, and ONL-thickening in areas with HRF. This predictive model achieved an AUC of 0.68 for nAMD and AUC of 0.80 for GA prediction. While outer retinal thinning leads to GA, volume, and size of drusen predicted conversion to nAMD, although features of both were often present at the same time. Furthermore, the model demonstrated a low prognostic value for genetic profiles [59], therefore prognostic algorithms often omit these features. Moreover, clinicians rarely investigate the genetic status of patients, as it often comes with a price and a long time until results can be ascertained. “Deep Sequence” is a hybrid sequential prediction model for calculating the risk of exudation within 3 and 21 months in non-exudative AMD. It is based on radiomics-engineered OCT imaging features, demographic, and visual factors combined with a RNN and it achieved high performances on cross-validation. However, testing on an external dataset, the model showed a decrease in performance for 21-month prediction [64]. Another OCT-based fully autonomous algorithm called DeepGAze was developed to predict the risk for conversion to GA within 1 year using a position-aware CNN with proactive pseudointervention [65]. Algorithms predicting the time of conversion are beneficial for clinicians as they can plan check-ups accordingly, which can lower the burden for patients, clinicians, and healthcare systems.

### Advanced AMD

#### Geographic atrophy

GA is a chronically progressive degeneration of the choriocapillaris, the photoreceptors, and the RPE in the macula causing irreversible vision loss [66]. FAF was the main imaging modality in GA demarcating hypoautofluorescent areas of RPE loss through the detection of mainly lipofuscin and melanolipofuscin [67–69]. Additionally, NIR enhances the borders of the hyperreflective atrophy by using a longer wavelength, which changes interference with luteal pigments avoiding impacts of media opacities and foveal pigment. However, a thick choroid or hyperreflective lesions (e.g., crystalline drusen) can compromise the contrast. Limiting factors of FAF include media opacities and the absorption of blue light by the foveal pigment, which limits the assessment of foveal sparing [70]. Therefore, detecting small changes in the progression of retinal diseases on FAF and NIR is challenging even for human experts. AI-based algorithms, however, can provide accurate results comparable to human performance [71–74]. BCVA is insufficient as a biomarker for disease progression in GA [75]. Either the fovea is spared and BCVA is hardly affected, or the fovea is depleted and the BCVA is severely impaired with only minor changes possible thereafter. Therefore, regulatory authorities recently approved structural endpoints for clinical trials in GA [2, 3]. FAF is a reliable tool to measure GA growth rate [33] and hypoautofluorescent areas correspond to scotomas in the visual field [76, 77], hence it has been used as primary endpoint in clinical trials [78, 79].

#### AI in assessing progression of GA in FAF

Early automated segmentation by image analysis software (Global Lab Image/2) demonstrated by Schmitz-Valckenberg et al. tends to underestimate atrophic areas in FAF [70]. However, intraobserver agreement and interobserver agreement were superior to manual delineation. This method was used to quantify atrophies in a longitudinal natural history study of GA [80]. Thereafter, Deckert et al. developed an integrated contrast-enhancing tool improving uneven illumination, which results in clearer borders of the atrophy [81]. Furthermore, follow-up visits were aligned by combining retinal vessels as landmarks compensating disturbances from scaling, shifting, and rotation [82]. These alignments are important for implementation in clinics as the quality of real-world images is frequently inferior to those from clinical trials. The RegionFinder (Heidelberg Engineering, Heidelberg, Germany) is a commercially available semiautomatic algorithm developed by two reading centers used to longitudinally delineate atrophy on FAF. Here, the examiner needs to manually adjust the contrast threshold and delineations [74], which is a time-consuming and more subjective task compared to fully automated algorithms and therefore not ideal for implementation in the real world. Hu et al. developed an ML-based algorithm using supervised pixel classification. In this method, the algorithm learns to delineate atrophy from calculating the likelihood of the neighboring pixel belonging to GA (supervised pixel classification), which achieved an accuracy of 0.94. Even though the algorithm achieved a good performance in the preliminary study, results from validation of this ML approach suggest that there is still room for improvement [83]. Fully automated methods using DL have been developed recently. A five-fold validated U-NET-based algorithm by Arslan et al. was developed to automatically segment GA on FAF achieving a dice similarity coefficient (DSC) of 0.98-9 using cross-validation without external validation [72]. Another algorithm introduced recently by Spaide et al. combining a U-NET and a Y-NET on FAF and NIR reached 0.89–0.92 DSC in a longitudinal assessment of GA growth similar to human segmentation. For the development of this algorithm only one dataset was used for training and validation, therefore the performance has not been tested in independent, heterogeneous external datasets [71]. All these different approaches resulted in good to excellent performances in quantifying GA size, providing benefits including precision, reproducibility, and time-effective assessments of individual patients or large datasets [71]. However, some of these studies reported the need for human interaction defining regions of interest and false positives or negatives [83]. These difficulties in FAF-based GA assessments impede the implementation of these algorithms into everyday clinical routine.

#### Predicting progression of GA in FAF

With novel therapies approved for patients with GA, observing changes in the speed of progression is important for the assessment of treatment efficacy. Since treatment slows progression [2, 3, 84], starting soon after conversion to GA would be beneficial. Furthermore, due to potentially limited resources with respect to caregivers and therapeutic agents, a high socioeconomic burden and potential therapeutic side effects, clinicians might have to select patients profiting most from therapy. Therefore, clinicians need to predict progression, which is a difficult task for humans. In FAF, differing progression rates based on multiple imaging patterns have been described. No abnormal FAF pattern or unifocal lesions had the lowest progression rates, whereas multifocal, banded, and diffuse patterns, especially the diffuse trickling pattern, were the forms with the fastest progression. Furthermore, other morphologic characteristics have been associated with faster disease progression, such as larger lesions, lesion location, and shape, e.g., multifocality [5, 80]. A large interindividual variability in atrophy growth rates impedes accurate prediction of disease progression of individuals [85]. With the help of the semiautomatic RegionFinder, a study demonstrated only ~24–39% of the variability in GA progression rates derived from FAF-based lesion area, circularity, perimeter, calliper diameters as well as FAF pattern, which were assessed automatically [86]. These results highlight the limited value of FAF-derived features, suggesting that part of the remaining variation can be explained by other systemic (e.g., genetic), or OCT-based biomarkers.

#### AI for assessment of GA progression in OCT

Today, OCT is the gold standard of GA diagnosis and management, providing three-dimensional visualization of retinal structures in high resolution. FAF-based quantification of atrophic lesions has shown high correlations to RPE loss on OCT [87]. Therefore, the equivalent biomarker for determining progression speed of atrophy on OCT is RPE loss, although other features visible on OCT give deeper insights into progression patterns in eyes with GA [88]. In cross-sectional B-scans, the RPE layer can be assessed manually or automatically by direct assessment of the RPE layer or the underlying hypertransmission [89]. Hypertransmission is defined as a hyperreflective signal in the choroid, which results from the loss of photoreceptors and the RPE leading to lacking light absorbance. Automatic segmentation of hypertransmission on OCT using a sub-RPE slab is an accurate and reproducible method for longitudinal GA assessment. A deep learning model trained on swept-source OCT images reached a symmetric dice coefficient of 0.88. The performance of this particular algorithm was limited due to faulty automated segmentation of retinal layers, particularly the Bruch’s membrane, by the instrument software. For this model, data augmentation from the deep learning algorithm randomly altered the training samples to improve the training capacities of neural networks. This method is used when only limited datasets are available, as reported in this study [90]. On OCT, not only the RPE, but also photoreceptors can be assessed through the EZ corresponding to the ellipsoid of the inner segments [91]. Recently, the loss of the EZ on OCT imaging has been recognized as important biomarker by regulatory authorities [92], since reduced sensitivity has been associated with loss of the inner segment ellipsoid [24, 93, 94]. Therefore, establishing validated algorithms segmenting these features automatically will be necessary in large datasets from clinical trials. In a post-hoc analysis of the FILLY trial, a phase II trial investigating the complement inhibitor Pegcetacoplan, validated deep-learning algorithms provided topographic maps of RPE and photoreceptor integrity loss from OCT B-Scans. A significant protective treatment effect on RPE loss was demonstrated, with an even higher impact on photoreceptor integrity loss [84]. In this study, the previously mentioned U-Net-based CNN model by Orlando et al. delineated the photoreceptors as EZ and IZ in cross-sectional B-scans calculating en face maps from voxel-level binary segmentations [14]. For RPE segmentations, a DL model by Lachinov et al. was used. A 3-dimensional-to-2-dimensional CNN was trained and five-fold validated to detect RPE loss on cross-sectional B-scans followed by visualization as binary en face map [95].

#### AI predicting progression of GA in OCT

**Fig. 3:**
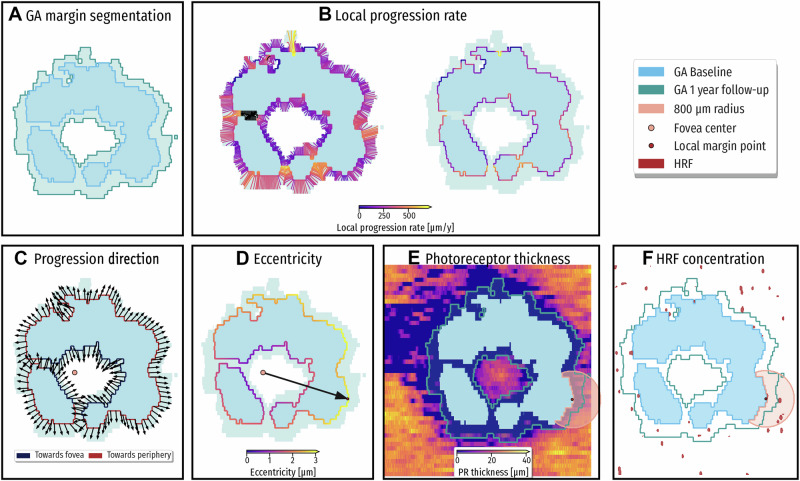
Spatio-temporal predictive model for GA progression. Spatio-topographic features for each GA margin point. The baseline GA area is shown in blue and 1-year progression is shown in green. **A** Automated segmentation of retinal pigment epithelium (RPE) loss determining GA at baseline and at 1 year. **B** Local progression rate (LPR) is color coded at each margin point to illustrate local progression activity. **C** The growth direction toward the fovea (blue margin) or toward the periphery (red margin). **D** For each GA margin point, the distance to the fovea is computed. **E** For each GA margin point, the mean photoreceptor (PR) thickness in the junctional zone is computed. **F** For each GA margin point, the hyperreflective foci (HRF) concentration is computed. Red dots in (**F**) mark locations of HRF in the retina. Reproduced with permission from Vogl et al. [5].

Observing the longitudinal growth of GA is important for patient management. Automated quantification on OCT is key in assessing therapeutic efficacy not only for RPE, but also for photoreceptors. An association between GA growth rates and the ratio between the area of photoreceptor integrity loss and RPE loss was found. Patients with higher ratios, meaning larger areas of photoreceptor integrity loss compared to RPE loss (PR loss/RPE loss ratio), presented with faster growth rates [84]. Furthermore, Vogl et al. developed a validated DL algorithm to predict topographic progression of GA by analyzing RPE loss, photoreceptor integrity and HRF in four steps (Fig. 3). CNN-based automated segmentations of GA lesions, photoreceptor layers, and HRF on OCT were conducted (step 1). Then, follow-up scans were registered to baseline images (step 2) followed by quantification of local progression rates and biomarkers in relation to atrophy borders (step 3). In a last step, the local GA progression rates were estimated using a biophysical growth model by calculating trajectories for each point around the margin of the atrophy (step 4). An association was found between higher progression rates and atrophic lesions closer to the fovea, HRF at the junctional zone and thinner photoreceptor layers. Still, there are few limitations within this study, derived mostly from the dataset. First, a relatively small number of B-Scans per OCT resulted in large spacing between slices. Therefore, small growth cannot be observed. To overcome this problem, a compound Poisson-gamma distribution was assumed, where zero has a positive value. Another challenge was a staircase effect due to the B-Scan slices, which was minimized with a higher smoothing factor in the interface propagation. Lastly, large, fast-growing GA lesions protruding beyond the 6 × 6 mm field of view cannot be assessed leading to a downward bias in estimations of the peripheral regions [5]. Another approach for predicting progression has been published by Anegondi et al. using multimodal imaging in a multitask approach. For this purpose, three algorithms were trained on FAF only, OCT only and both combined and all three achieved similar accuracies during testing. FAF and OCT-derived data are combined in a DL model to estimate GA lesion size and growth rates simultaneously as the slope of a linear fit on measurements of lesion area over 2 years [73]. The benefit of the multitask approach is a short training period, because one model can perform two tasks separately, at the same time. Overall, these tools could help in identifying progression patterns and speed for individual patients, which would support clinicians for the task of patient selection with respect to treatment.

### Neovascular AMD/exudative AMD

Ten to fifteen percent of patients with iAMD develop nAMD [93]. nAMD is characterized by disruption of the outer retina by proliferation of MNV and exudation of fluid into or under the neurosensory retina [96], which can be visualized as leakage on fundus fluorescein angiography and/or indocyanine angiography and was initially defined as occult or classic choroidal neovascularization. An OCT-based classification was established changing the nomenclature from choroidal neovascularization to MNV by international consensus and further subclassified based on lesion location: MNV type I includes neovascularization into the sub-RPE space; MNV type II comprises lesions above the RPE, while MNV type III refers to vessel growth from the deep capillary plexus towards the outer retina [97]. MNV types cause pathognomonic fluid accumulations of intraretinal fluid (IRF), subretinal fluid (SRF), and pigment epithelial detachments (PED) impairing visual function to different degrees [97].

#### Current treatment regimens and real-world outcomes

Anti-VEGF therapy was established as the gold standard treatment of nAMD [98]. Although monthly regimes were initially considered to have the maximum efficacy for visual acuity benefits [99], they pose a risk of overtreatment for a majority of patients, resulting in a higher burden for patients as well as caretakers [100, 101]. Hence, pro re nata (PRN) regimes treating disease activity defined as OCT-based reoccurrence of exudation as well as treat-and-extend regimes with proactive interval extension from monthly to bimonthly and further to every 12 and 16 weeks based on individualized intervals between treatments according to patient needs were proposed [102, 103].

However, both regimes achieve inferior success in real-world clinical practice compared to clinical trials, most likely due to undertreatment in suboptimal and/or disrupted treatment intervals [104], as well as concurrent development of outer retinal atrophy and fibrosis [105, 106]. While fibrosis development affected 45.5% of eyes after two years in the CATT trials [107], more recent real-world analyses estimated a cumulative incidence of fibrosis development of 62.7% after 10 years of PRN treatment in the real world [108]. Yet, both treatment regimens are dependent on OCT-based monitoring of disease activity, generally defined as recurrence or persistence of SRF and/or IRF [99].

#### Potential benefits from fluid volume quantification

Consequently, it became of high clinical relevance to objectively quantify fluid compartments in OCT volumes. Waldstein et al. established a method of translating fluid segmentation on OCT B-scans to volumetric information, whereas manual annotation of whole OCT volumes for this purpose took up to 15 h [109]. Therefore, manual annotation and subsequent quantification of the three vision-relevant fluid compartments, IRF, SRF, and PED in full OCT volumes, consisting of 97, 128, or 193 B-scans as provided by the most commonly used OCT manufacturers, is not feasible in busy clinical practice. With recent breakthrough advances in AI, there is ongoing development of ML and DL algorithms to overcome this burden and facilitate treatment monitoring based on quantifiable metrics as compared to scrolling through OCT volumes to qualitatively look for fluid presence or absence.

#### Automated quantification of fluid volume in SD-OCT

**Fig. 4:**
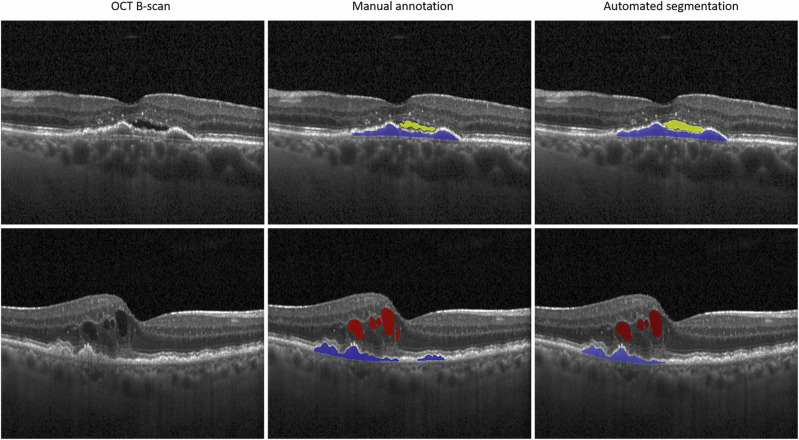
Automated quantification of retinal fluid compartments. Automated segmentation of intraretinal fluid (red), subretinal fluid (yellow) and pigment epithelium detachment (blue) in Spectral-Domain Optical coherence tomography using the Vienna Fluid Monitor (Version 2.5, Retinsight, Vienna, Austria) compared to manual annotation (second and third column).

ML-algorithms were developed to measure disease activity via quantification of retinal fluid compartments [110]. Concurrent advances in DL allowed for implementation of a DL model for image segmentation with automated fluid volume quantification, as well as probability metrics for classification to different diseases [111]. Pioneering work by Schlegl et al. demonstrated that DL-algorithms are suitable for automated fluid volume quantification in SD-OCT volumes in Cirrus (Carl Zeiss Meditec, Dublin, CA, USA) and Spectralis (Heidelberg Engineering, Heidelberg, Germany) OCT scans [112]. This DL-algorithm, further developed into the Fluid Monitor (RetInSight, Vienna, Austria) was validated in big datasets consisting of 11 127 eyes (Fig. 4) [113]. Nowadays, DL-algorithms are continuously developed for the purpose of automated image segmentation and better understanding of disease morphology for their prognostic value and functional outcomes during follow-up by various research groups [114, 115]. Reported limitations include the use of different OCT devices throughout big datasets [114], as well as subjectivity of human expert reference for algorithm training, especially for IRF [115].

Suboptimal outcomes in real-world clinical practice and novel developments in OCT technology combined with the possibilities of automated quantification of fluid volume led to concurrent efforts to allow home-based monitoring with home OCT devices. With the introduction of the Home OCT (Notal Vision Home OCT, Notal Vision Inc., Manassas, VA, USA), pivotal teleophthalmology concepts were developed, combining self-sufficient patient monitoring with previously developed fluid segmentation algorithms [116, 117]. Novel insights into disease activity with fast treatment response and personalized treatment regimens are expected to rise from this recent progress in home monitoring. However, these methods seek further validation in bigger cohorts during longer follow-up and conceptualization in big clinical trials [116]. Also, clinical applicability of these tools in real-world elderly cohorts of nAMD patients’ needs to be established.

#### AI for quantification of disease activity and correlation to function

Post-hoc analyses of the HARBOR and FLUID trials established that IRF in the central mm is associated with worse visual outcomes, whereas SRF volume in the central mm did not impact visual acuity negatively [118]. Consistent with this result, stronger association between IRF with visual acuity compared to SRF were reported in a different database by Moraes et al. [119]. The prognostic value of each fluid compartment during follow-up was further analyzed in a post-hoc analysis of the HAWK and HARRIER trials [120]. Patients with high IRF, SRF, and PED volume at baseline experienced lower visual acuity levels during follow-up [120]. Overall, these automated analyses of fluid volume confirmed early findings from post-hoc analyses of IRF presence vs. absence in the CATT trial, where IRF was described as a strong biomarker for worse visual outcomes after 2 years of treatment [121]. Chakravarthy et al. demonstrated an impact of IRF, SRF and PED volume fluctuations during anti-VEGF treatment with worse visual acuity outcomes in groups with higher volume fluctuations after 2 years [122]. Exploratory post-hoc analyses of the OSPREY trial included automated quantification of subretinal hyperreflective material (SHRM) and EZ attenuation in addition to IRF, SRF, and PED volume and demonstrated an association between visual functional outcome and SHRM and EZ attenuation during follow-up [123]. Limitations of this analysis included that the EZ attenuation was defined at the level of the EZ-RPE compartment, not the EZ band and application on one device.

Implementation of DL-based fluid segmentation in real-world data from the Free Retinal Blindness! Registry in Spain confirmed previous findings from post-hoc analyses of clinical trials: baseline foveal IRF was associated with worse visual acuity at baseline and after 1 year of follow up [114]. Reiter et al. conducted an extensive analysis in another real-world dataset and concluded that patients with high IRF volume had significantly lower visual acuity after four years, as well as a higher number of injections. Furthermore, patients with high fluid volume after the initial three anti-VEGF injections with short intervals (loading dose) needed more treatment during follow-up. After 4 years, atrophy development occurred in 40% of eyes, whereas fibrosis developed in 23% of eyes [124].

#### Prediction of AMD progression and treatment outcomes

Due to high interindividual variabilities regarding fluid activity and functional outcomes, prediction of treatment response and treatment need continuously gained clinical relevance. Pivotal work on the dataset from the HARBOR trial reported the potential of ML-algorithms to predict visual acuity from baseline Cirrus SD-OCT volumes [125]. An ML algorithm based on random forest regression was trained with OCT biomarkers segmented by fully automated fluid (IRF, SRF, PED) and layer algorithms as input and visual acuity at baseline as target variable to predict visual acuity at month 12 [125]. The most relevant prognostic biomarker for visual acuity at month 12 was baseline visual acuity and IRF in the central mm, with better predictive value if the parameters at month three were included to the model (*R*^2^ = 0.70). Subsequently, a similar method was used on the Moorfields Eye Hospital AMD database of 137 379 OCT volumes [126]. The prediction model was trained based on automated segmentation of retinal layers and fluid compartments in newly diagnosed eyes after loading dose and implemented to 3DOCT2000 SD-OCT (Topcon, Tokio, Japan) scans. The impact of IRF, SRF, PED, SHRM, and HRF for prediction of visual acuity after the first three injections and at month 12 was examined. Model accuracy reached *R*^2^ = 0.53 based on baseline visual acuity and the above-mentioned OCT biomarkers and improved after addition of treatment response to the model (*R*^2^ = 0.72 and 0.63) [126]. A deep-learning model for prediction of treatment requirement during a 2-year follow-up was trained and validated on a dataset of OCT volumes from 423 patients in a PRN treatment regimen with 281 patients in the test set and 69 in the validation set [127]. The architecture is based on densely connected neural network (DenseNet) and an RNN, that is trainable end-to-end and achieved an AUC of 0.85 in classification of high and low anti-VEGF treatment requirement groups [127].

Mares et al. applied AI-algorithms to Spectralis SD-OCT to predict visual acuity, atrophy and fibrosis development in a real-world cohort and confirmed previous findings on baseline visual acuity as the most predictive biomarker for vision after 12 months. Model prediction of the number of injections reached an AUC of 0.77. An AUC of 0.70 and of 0.74 was achieved for predicting development of macular atrophy and fibrosis during a 4-year follow-up, respectively [128].

GANs for synthetic post-therapeutic OCT image reconstruction represent another field of DL-based treatment prediction [129]. Synthetic OCT images were compared to the actual post-therapeutic Topcon swept source-OCT image acquired after 4–6 weeks and evaluated for quality defined as qualification for clinical interpretation and accuracy of macular classification (accuracy 0.85) and wet-to-dry conversion (accuracy 0.81) [129]. However, to date this method was only established during short-term follow-up and the clinical meaning for long-term outcomes and image prediction is yet to be established.

## Limitations

Automated algorithms clearly have benefits, although there are a few limitations to consider. The quality and diversity of datasets for training and validation of algorithms need to represent the real population. Therefore, development often demands large datasets to prevent underrepresentation of specific patient characteristics such as ethnicity. Another aspect to consider is the interpretability of AI-based decisions. Especially with unsupervised learning, it remains unclear how the algorithm achieves its result, a so-called “blackbox” model [8]. Furthermore, algorithms constantly need to adapt to imaging protocols and varying image quality in the real world in contrast to datasets used for training. There are few universally applicable algorithms, due to the introduction of novel imaging modalities and devices. Further aspects include safety regarding patient privacy and data security, particularly when data from commercially available AI-based image analysis software is sent to a cloud to be processed. Security systems, data storage, and computational power can be a financial burden and these commercially available algorithms often come with a price as well. Although AI has its share of limitations, implementation holds promise for the future when keeping these aspects in mind.

## Future directions and conclusion

AI has entered clinical practice in the diagnosis and management of all stages of AMD. Identification of patients with early signs of progression and planning of individual monitoring visits is important to target the earliest time for intervention. Advances in OCT technology have greatly facilitated the implementation of anti-VEGF therapy in nAMD by visualizing treatment response. However, treatment options for non-exudative AMD do not come with functional improvement for the patient. The age of AI is therefore even more important to detect and quantify these subclinical changes and evaluate every possible voxel on the OCT, a task that is not feasible for human experts. In addition, new tasks, such as predicting disease progression, have never been done by an ophthalmologist. However, the AI reproduces what it has learned and outperforms clinicians in terms of time savings and accessibility of knowledge.

Validation of AI models, including validation on external and diverse datasets, will be an ongoing field for years to come. The availability of AI models to support clinicians in their daily tasks is becoming more comprehensive. Models for disease screening using the vast availability of imaging devices will gain momentum in the near future, and risk stratification, including functional prognosis, may become routine counseling information. The synergy of the large amount of data generated by OCT and the AI models to analyze this data will remain an essential new part of the ophthalmologist’s practice, guiding decision-making with quantifiable and objective evidence for patient-tailored and disease-targeted medicine.
